# TGF-Beta Negatively Regulates the BMP2-Dependent Early Commitment of Periodontal Ligament Cells into Hard Tissue Forming Cells

**DOI:** 10.1371/journal.pone.0125590

**Published:** 2015-05-13

**Authors:** Takanobu Kawahara, Motozo Yamashita, Kuniko Ikegami, Tomomi Nakamura, Manabu Yanagita, Satoru Yamada, Masahiro Kitamura, Shinya Murakami

**Affiliations:** Department of Periodontology, Graduate School of Dentistry, Osaka University, 1–8 Yamadaoka, Suita-Osaka 565–0871, Japan; University of California Davis, UNITED STATES

## Abstract

Transforming growth factor beta (TGF-β) is a multi-functional growth factor expressed in many tissues and organs. Genetic animal models have revealed the critical functions of TGF-β in craniofacial development, including the teeth and periodontal tissue. However, the physiological function of TGF-β in the periodontal ligament (PDL) has not been fully elucidated. In this study, we examined the roles of TGF-β in the cytodifferentiation of PDL cells using a TGF-β receptor kinase inhibitor, SB431542. Mouse PDL cell clones (MPDL22) were cultured in calcification-inducing medium with or without SB431542 in the presence or absence of various growth factors, such as bone morphogenetic protein (BMP)-2, TGF-β and fibroblast growth factor (FGF)-2. SB431542 dramatically enhanced the BMP-2-dependent calcification of MPDL22 cells and accelerated the expression of ossification genes *alkaline phosphatase* (*ALPase*) and *Runt-related transcription factor* (*Runx*) *2* during early osteoblastic differentiation. SB431542 did not promote MPDL22 calcification without BMP-2 stimulation. The cell growth rate and collagen synthesis during the late stage of MPDL22 culture were retarded by SB431542. Quantitative reverse transcription polymerase chain reaction analysis revealed that the expressions of *Smurf1* and *Smad6*, which are negative feedback components in the TGF-β/BMP signaling pathway, were downregulated in MPDL22 cells with SB431542 treatment. These results suggest that an endogenous signal from TGF-β negatively regulates the early commitment and cytodifferentiation of PDL cells into hard tissue-forming cells. A synthetic drug that regulates endogenous TGF-β signals may be efficacious for developing periodontal regenerative therapies.

## Introduction

One method of regenerating lost periodontal tissue requires the stimulation of somatic stem cells within the periodontal ligament (PDL) to differentiate into osteoblasts and cementoblasts. Cytokine therapy is an efficient procedure for stimulating these cell types [1]. Current cytokine therapies using a single cytokine with scaffold molecules may not be as efficient for treating large tissue defects [2,3]. A more detailed understanding of how multiple growth factors orchestrate cellular communications during the development and regeneration of periodontal tissue is needed to improve this therapeutic approach.

Transforming growth factor beta (TGF-β) is a multi-potential growth factor expressed in many tissues that affects a wide range of physiological phenomena such as cell proliferation, differentiation, migration, adhesion, and induction of extracellular matrix (ECM) synthesis [4]. TGF-β superfamily molecules transmit their signals through both types I and II serine/threonine kinase receptors [5]. The type II receptors are constitutively activated and they phosphorylate intracellular domain of type I receptors upon ligand ligation. There are seven type I receptors, named activin receptor-like kinases (ALK) 1–7 in mammals. TGF-β variants and activins bind to ALK-1, -4, and -5, whereas bone morphogenetic proteins (BMPs) bind to ALK-2, -3, and -6. ALK-7 transduces nodal signaling. Type II receptors activate these type I ALK receptors. Once activated, the ALK receptors transduce signals from the cell surface and cytosolic compartments to the nucleus to activate targeted genes via Smad proteins [6,7,8,9,10].

TGF-β is also abundant in hard tissue, and contributes to the regulation of hard tissue formation during development and tissue remodeling. TGF-β is stored in alveolar bone and in the connective tissue of the periodontium [11, 12], and is reported to be the most prominent cytokine distributed throughout the PDL. Interestingly, the expression level of TGF-β in PDL tissue is greater than that in pulp or alveolar bone [13]. This strongly suggests the involvement of TGF-β in the homeostasis, wound healing, and regeneration of periodontal tissues. However, the function of endogenous TGF-β in PDL cells has not been fully clarified.

In this study, we examined whether endogenous or exogenous TGF-β regulated the differentiation of PDL cells into hard tissue-forming cells using SB431542 [14], a small molecule inhibitor of TGF-β signal. SB431542 is a cell-permeable small chemical compound that inhibits ALK-4, -5 and -7 kinase activity by specifically antagonizing ATP binding to the intracellular kinase domain. SB431542 treatment enhanced the formation of mineralized tissue in BMP-2-stimulated MPDL22 cells. SB431542 affected the cytodifferentiation of MPDL22 cells during the early ossification phase and down-regulated collagen synthesis during the late ossification phase. These results suggested that TGF-β has opposing functions during the process of hard tissue formation by PDL cells. Thus, SB431542 promotes the BMP-2-induced cytodifferentiation of MPDL22 cells by inhibiting the endogenous signal from TGF-β along the osteoblastic lineage. Topical application of a TGF-β inhibitor, such as SB431542, in combination with recombinant growth factors, may be an efficient treatment method for the periodontal regeneration of large tissue defects and could increase the indicated applications of current cytokine therapies.

## Results

### Expression of the TGF-β/BMP receptor and intracellular signaling components in MPDL22 cells

**Fig 1:**
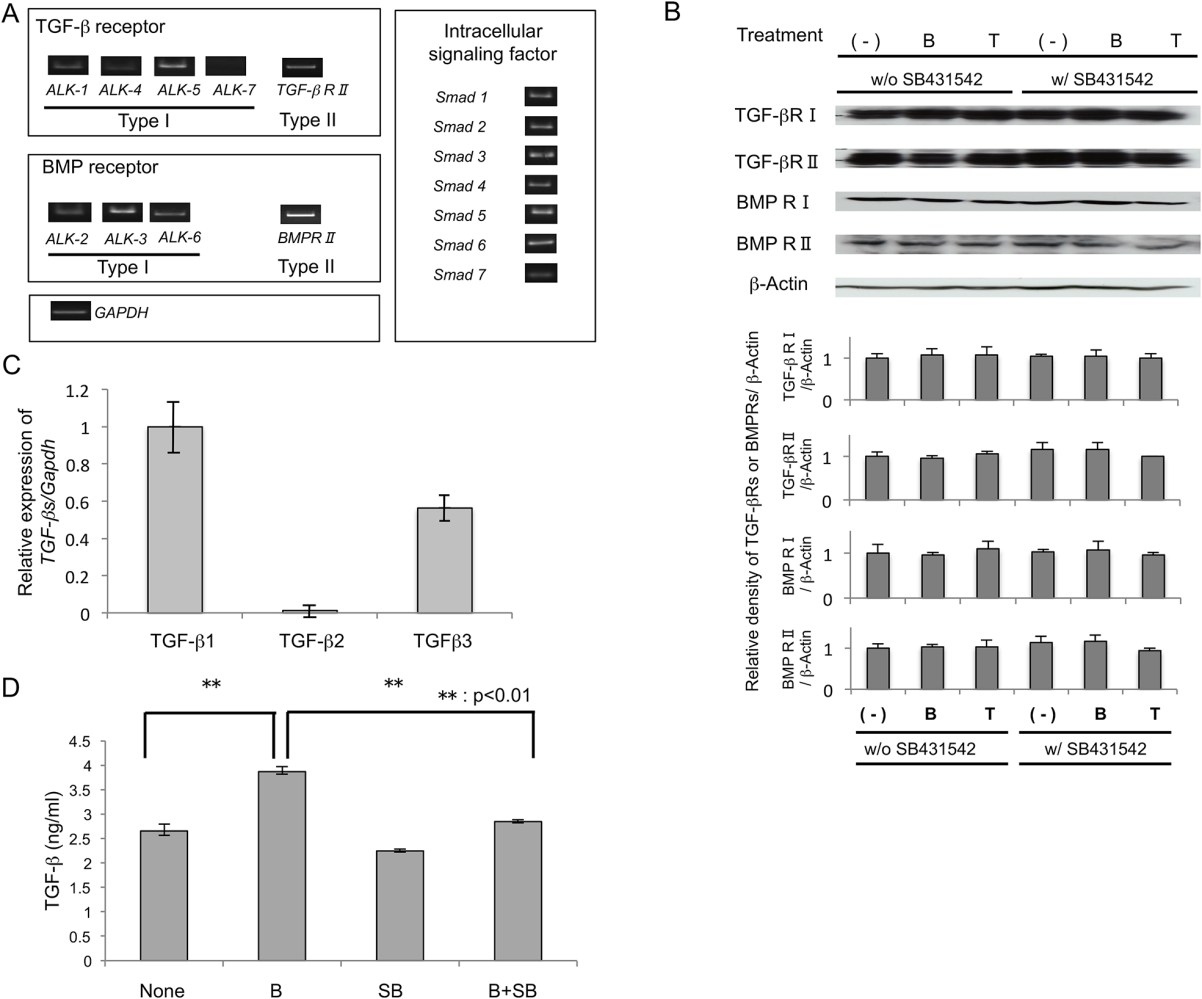
Expression of TGF-β/BMP receptor and Smads in MPDL22 cells. (A) Semiquantitative RT-PCR analysis of the expression of TGF-β receptor genes *ALK-1*, *-4*, *-5*, *-7*, and *TβRII*, and BMP receptor genes *ALK-2*, *-3*, *-6*, *BMPR2*, and *Smad1–7*. Human glycerralaldehyde-3-phosphate dehydrogenase (*GAPDH*) was used as an internal control. (B) Western blotting analysis of TGF-β/BMP receptor induced by TGF-β (4 ng/mL) or BMP-2 (50 ng/mL) in the presence or absence of SB431542 (10 μM). Protein levels of TGF-β receptor I (TGF-βRI), TGF-β receptor II (TGF-βRII), BMP receptor I (BMPRI), and BMP receptor II (BMPRII) were measured. β-actin was used as a protein loading control. Quantitative analysis is shown as the relative ratios of TGF-β or BMP receptors I/II and β-actin by densitometric analysis. Values represent the mean ± SD of 3 independent assays. (-): control; B: BMP-2; T: TGF-β. (C) The relative quantification of *TGF-β1*, *TGF-β2*, and *TGF-β3* mRNAs in MPDL22 cells by RT-qPCR. Quantitative mRNA values were normalized to the amount of *GAPDH* mRNA. (D) TGF-β production from MPDL22 cells. Protein expression levels of TGF-β were examined by ELISA. Culture supernatants of MPDL22 cells were aspirated after 24 h of culture with or without BMP-2 (50 ng/mL) and SB431542 (10 μM). B: BMP-2; SB: SB431542. **: p<0.01 vs the BMP-2 stimulated group.

We first examined the expression levels of TGF-β/BMP receptor and Smad intracellular signaling components in PDL cells (Fig 1A). Reverse-transcription polymerase chain reaction (RT-PCR) analysis demonstrated a moderate expression of ALK-3 and -5, whereas the expression of ALK-1, -2, and -6 was low and that of ALK4 was negligible. The expression levels of both TGF-β and BMP type II receptor were comparable (Fig 1A). The mRNA expression of Smads 1–7 were measured by RT-PCR, and the mRNA expression levels of each Smad were comparable in MPDL22 cells (Fig 1A). The expression of these receptors for TGF-β and BMP were confirmed at the protein level by western blotting. The effect of TGF-β and BMP stimulation on the receptor expression in MPDL22 cells at the protein level with or without SB431542 combined with the growth factor treatment was negligible (Fig 1B).

### Endogenous TGF-β production from MPDL22 cells

Expression of TGF-β1, TGF-β2, and TGF-β3 has been reported in the periodontal tissue of mice [11]. To confirm this, we examined the expression of TGF-β1, TGF-β2, and TGF-β3 in MPDL22 cells by quantitative reverse transcription polymerase chain reaction (RT-qPCR). The expression level of TGF-β1 was higher than that of TGF-β2 and TGF-β3 (Fig 1C). We also showed by enzyme-linked immunoabsorbent assay (ELISA) that MPDL22 cells constitutively secreted TGF-β1 (approximately 2.5 ng/mL in the culture supernatants of each well seeded with 4×10^5^ cells) (Fig 1D). BMP-2 enhanced the production of TGF-β from MPDL22 cells, whereas SB431542 treatment combined with BMP-2 suppressed the BMP-2-induced TGF-β elevation (Fig 1D). This suggested that SB431542 inhibited BMP-2-enhanced TGF-β production by repressing the autocrine endogenous TGF-β signaling.

### Effects of SB431542 on TGF-β signaling in MPDL22 cells

**Fig 2:**
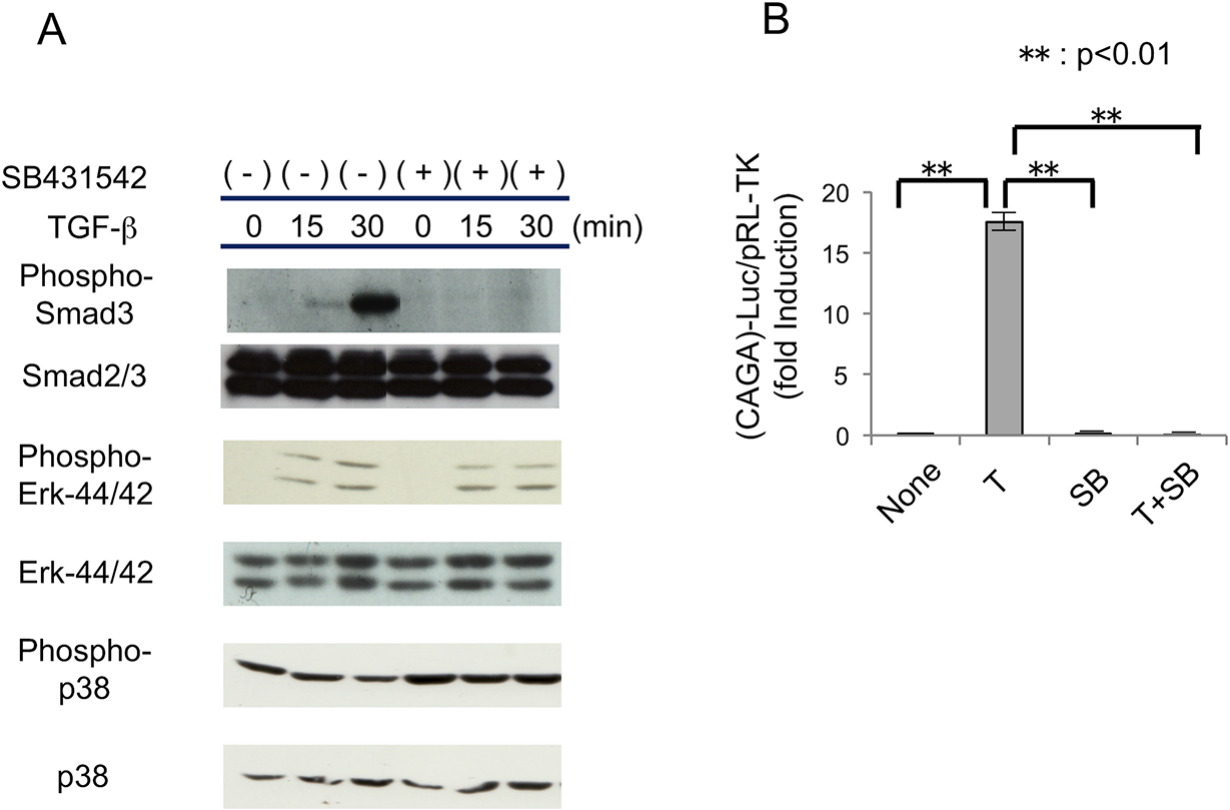
Effects of SB431542 on the TGF-β/Smad transcriptional responses in MPDL22 cells. (A) Activation of Smad3, Erk, and p38 induced by TGF-β (4 ng/mL) with or without pretreatment with SB431542 (10 μM). Phosphorylation levels and protein levels were determined by western blotting. (B) Promoter activity of TGF-β responsive gene *PAI-1*. MPDL22 cells were transfected with (*CAGA*)_12_-Luc reporter plasmid as indicated. Twenty-four hours after transfection, cells were treated with TGF-β (4 ng/mL), SB431542 (10 μM) or both overnight. (-): control; B: BMP-2; T: TGF-β; SB: SB431542. **: p<0.01 vs the TGF-β stimulated group.

We next examined the effects of SB431542 on TGF-β signaling in MPDL22 cells by western blotting using a specific anti-Smad3 antibody. Immunoblot data showed that pretreatment with 10 μM SB431542 completely inhibited the Smad3 phosphorylation induced by 4 ng/mL TGF-β compared with dimethyl sulfoxide alone (Fig 2A). In contrast, 10 μM SB431542 did not affect the phosphorylation status of Erk or p38, which are involved in the Smad-independent TGF-β signaling pathway [15]. We then confirmed the effects of SB431542 on the TGF-β signaling pathway at the transcriptional level using a luciferase assay with a reporter construct involving the Smad3 binding motifs in the *PAI-1* gene promoter 12X(*CAGA*)-Luc [16]. Here, 10 μM SB431542 abolished the transcriptional activities induced by 4 ng/mL TGF-β (Fig 2B). These data suggest that 10 μM SB431542 specifically inhibited Smad-dependent TGF-β in MPDL22 cells. Thus, we use 10 μM of SB431542 to inhibit TGF-β fully in the following experiments.

### Effects of SB431542 on BMP-2-induced hard tissue formation in MPDL22 cells

**Fig 3:**
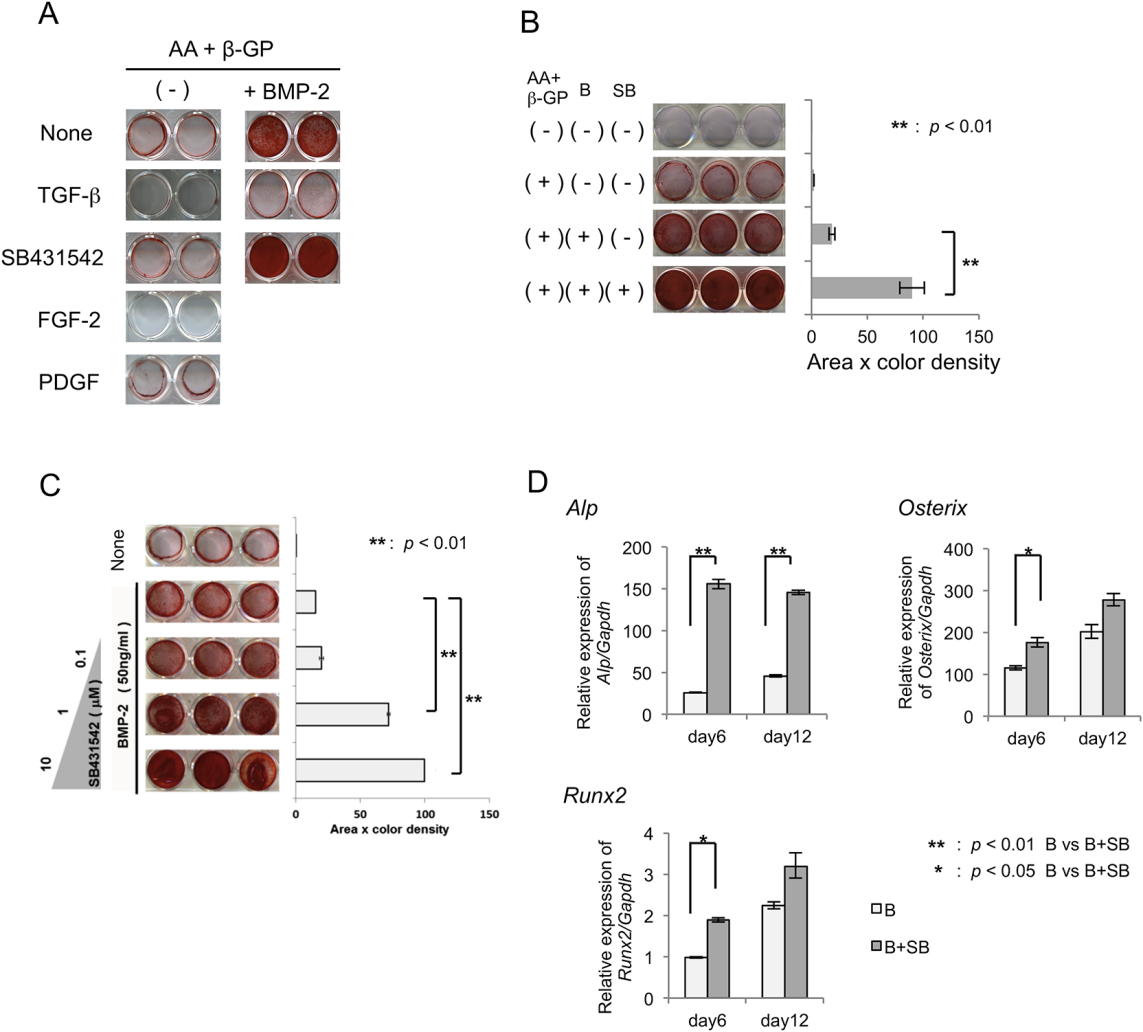
Effects of SB431542 on mineralized nodule formation by MPDL22 cells. (A) Osteogenic differentiation of MPDL22 cells was induced by culture in mineralization inducing medium with or without BMP-2 (50 ng/mL), FGF-2 (50 ng/mL) and PDGF-BB (20 ng/mL) in the presence or absence of TGF-β (4 ng/mL) and SB431542 (10 μM). Calcified nodule formation was determined at day 12 by Alizarin red staining. (B) Quantification of calcified nodule formation by MPDL22 cells induced by BMP-2 in the presence or absence of AA (50 mg/mL) plus β-GP (50 mM), BMP-2 (50 ng/mL) and SB431542 (10 μM). Densitometric analysis was applied to the scanned culture plate images at day 12. Positive scores were calculated by multiplying the stained area by its Alizarin red staining color density. B: BMP-2; SB: SB431542. **: p<0.01 vs BMP-2. (C) The effects of various concentrations of SB431542 on the mineralized nodule formation by MPDL22 cells. **: p<0.01 vs BMP-2. Quantification of the calcified nodule formation by BMP-2-stimulated MPDL22 cells in the presence of β-GP (50 mM) plus AA (50 mg/mL) with or without SB431542 (0.1, 1.0, and 10 μM). (D) The relative quantification of *ALP*, *Runx2*, and *Osterix* mRNAs during osteogenic differentiation of MPDL22 cells by BMP-2 (50 ng/mL) after treatment with or without SB431542 (10 μM) for 2 days. MPDL22 cells were harvested at days 6 and 12 and the isolated mRNA was assessed by RT-qPCR. Quantitative mRNA values were normalized to the amount of *GAPDH* mRNA. **: p<0.01 vs BMP-2; *: p<0.05 vs BMP-2.

Next, we examined the effects of SB431542 on the hard tissue-forming ability of MPDL22 cells. We previously reported that MPDL22 cells differentiated into hard tissue-forming cells, such as osteoblasts and cementoblasts, during long-term culture with ascorbic acid (AA) and β-glycerophosphate (β-GP) [17]. As shown in Fig 3A, BMP-2 induced calcified nodule formation in MPDL22 cells at day 12. In contrast, stimulation with TGF-β or FGF-2 inhibited the formation of calcified nodules induced by the mineralization-inducing medium (AA + β-GP). Although SB431542 alone slightly induced mineralization in MPDL22 cells in this system, SB431542 dramatically enhanced BMP-2-induced calcified nodule formation. This suggested that the inhibition of endogenous TGF-β function accelerated the calcified nodule formation by BMP-2. To confirm this, we evaluated the effects of SB431542 at different concentrations on MPDL22 cell calcification in the presence of BMP-2. As shown in Fig 3C, the addition of SB431542 up to 10 μM increased the calcified nodule formation in a dose-dependent manner. Density analysis of scanned images of Alizarin staining showed that 10 μM SB431542 treatment increased the BMP-2-dependent mineralized nodule formation by over 4-fold (Fig 3B and 3C). RT-qPCR analysis revealed that the BMP-2 dependent expression of ossification-related genes *ALP*, *Runx2* and *Osterix* were increased at days 6 and 12 in the presence of SB431542. These results suggested that SB431542 stimulated the BMP-2-induced osteoblastic differentiation of MPDL22 cells (Fig 3D).

### Effects of SB431542 on the early ossification of BMP-2-stimulated MPDL22 cells

**Fig 4:**
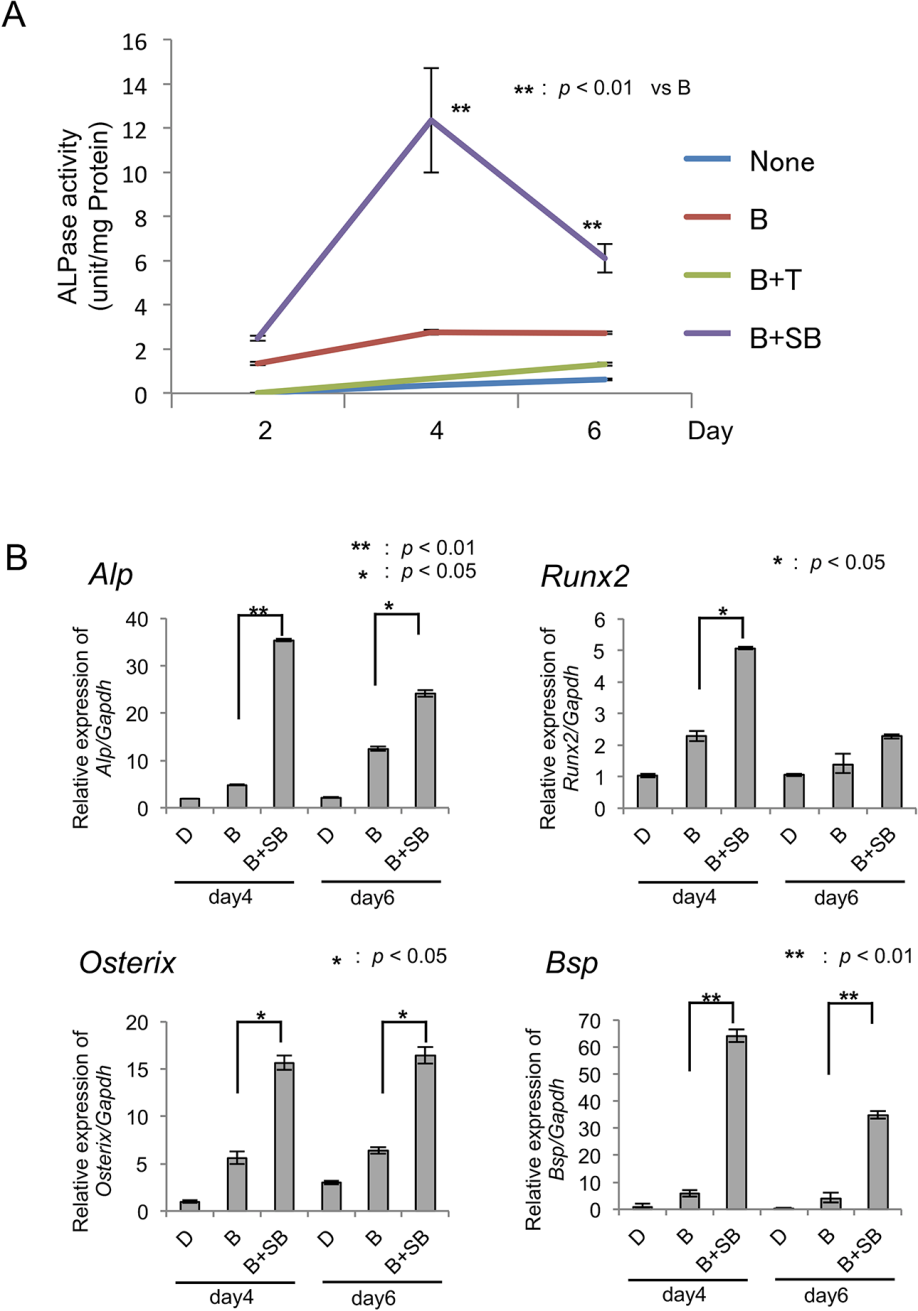
SB431542 treatment on ALP activity and expression of osteoblastic differentiation-related genes in MPDL22 cells. (A) MPDL22 cells were cultured in mineralization-inducing medium in the presence or absence of BMP-2 (50 ng/mL), TGF-β (4 ng/mL) and SB431542 (10 μM). MPDL22 cells were harvested at the indicated time points. ALPase activity was determined as described in the methods section. Activity in U/mg protein for the cell lysates is shown. **: p<0.01 vs BMP-2. (-): control; B: BMP-2; T: TGF-β; SB: SB431542. (B) Relative quantification of *ALP*, *Runx2*, *Osterix* and *BSP* mRNA expression levels was performed after 4 and 6 days of MPDL22 cell culture in the mineralization inducing medium with or without BMP-2 (50 ng/mL) and SB431542 (10 μM). D: AA plus β-GP; B: BMP-2; SB: SB431542.**: p<0.01 vs BMP-2; *: p<0.05 vs BMP-2.

To determine the cellular and molecular mechanisms by which SB431542 influences MPDL22 ossification, we examined the effect of SB431542 on ALPase activity (Fig 4A). When SB431542 was added, MPDL22 cells exhibited enhanced BMP-2-induced ALPase activity. We then examined the expression profiles of osteoblastic marker genes in MPDL22 cells. SB431542 treatment enhanced the mRNA levels of *ALP*, *Runx2* and bone sialoprotein (*BSP*) in BMP-2-stimulated MPDL22 cells. This increase in mRNA expression induced by SB431542 treatment was more apparent at day 4 than on day 6. The expression level of *Osterix*, which is a marker gene for the mature ossification process, showed similar levels in the presence or absence of SB431542 treatment when combined with BMP-2 stimulation in MPDL22 cells (Fig 4B). SB431542 treatment may alter the effects of BMP-2 on osteoblastic differentiation in MPDL22 cells by accelerating the mineralization process.

### Effects of SB431542 on the ossification of MPDL22 cells in different culture periods

**Fig 5:**
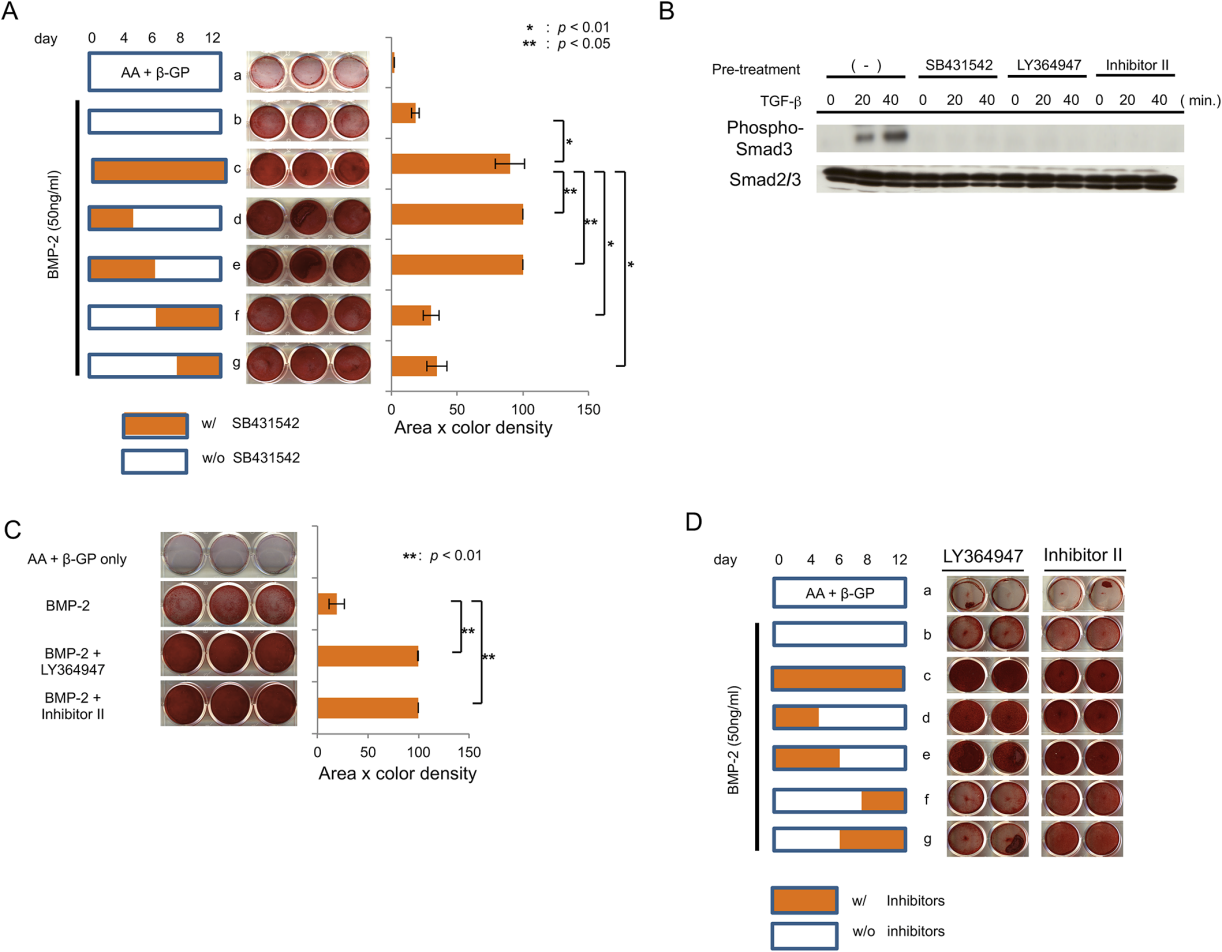
Effects of BMP-2 and SB431542 treatment at different periods during osteoblastic differentiation of MPDL22 cells. (A) MPDL22 cells were cultured in mineralization-inducing medium in the presence or absence of BMP-2 (50 ng/mL). SB431542 (10 μM) was also added to the culture at different times as indicated in the left panel. The middle panel shows Alizarin red staining at day 12. The right panel shows the quantification by densitometric analysis of the Alizarin red staining. **: p<0.01 vs BMP-2 + SB431542 (0–12); *: p<0.05 vs BMP-2 + SB431542 (0–12). (B) The effects of other classes of TGF-β receptor inhibitor, LY364947 and Inhibitor II, on osteoblastic differentiation in MPDL22 cells. Activation of Smad3 induced by TGF-β (4 ng/mL) with or without pretreatment by LY364947 (10 μM) or Inhibitor II (10 μM) was measured. Phosphorylation and protein levels of Smad3 were determined by western blotting. (C) LY364947 (10 μM) and Inhibitor II (10 μM) were added to the culture of MPDL22 cells during BMP-2-induced osteogenic differentiation. The right panel shows the quantification by densitometric analysis. **: p<0.01 vs BMP-2 (D) MPDL22 cells were cultured in mineralization inducing medium in the presence or absence of BMP-2 (50 ng/mL). LY364947 (10 μM) or Inhibitor II (10 μM) was also added to the culture at different times as indicated in the left panel. The middle panel shows Alizarin red staining at day 12.

The data in Fig 4 suggest that the effects of SB431542 should be different at each stage of differentiation in MPDL22 cells during long-term culture. To examine this, we treated BMP-2-stimulated MPDL22 cells with SB431542 at various time points to block endogenous TGF-β signaling. BMP-2 alone slightly increased the Alizarin staining of MPDL22 cells, and treatment with SB431542 throughout the 12 days resulted in the largest increase in Alizarin staining. In contrast, treatment with SB431542 only during the later phases of ossification (days 6–12) showed a smaller increase. Treatment with SB431542 during the early phase (days 0–4) of ossification resulted in more Alizarin staining than treatment only in the later phases (Fig 5A). These results suggested that the regulation of TGF-β signaling during the osteoblastic differentiation of MPDL22 cells is more important in the early phase than in the maturation phase.

### Effects of other TGF-β receptor inhibitors on the osteoblastic differentiation of MPDL22 cells

To confirm the study hypothesis, we examined the effects of other types of TGF-β receptor inhibitors. LY364957 and Inhibitor II inhibit TGF-β type I receptor kinase activity in the same manner as SB431542. Both of these inhibitors function by competing with ATP binding for the intracellular kinase sites of TGF-β type I receptors [18, 19]. As shown in Fig 5B, application of these inhibitors abolished the TGF-β (4 ng/mL)-induced phosphorylation of Smad3, similar to that with SB431542. In addition, they significantly enhanced the BMP-2-stimulated ossification of MPDL22 cells (Fig 5C). Similar to that seen with SB431542, the inhibition of TGF-β signaling by these drugs was most effective during the early phase of ossification of BMP-2-stimulated MPDL22 cells (Fig 5D).

### Effects of SB431542 on collagen synthesis in BMP-2-induced ossification culture of MPDL22 cells

**Fig 6:**
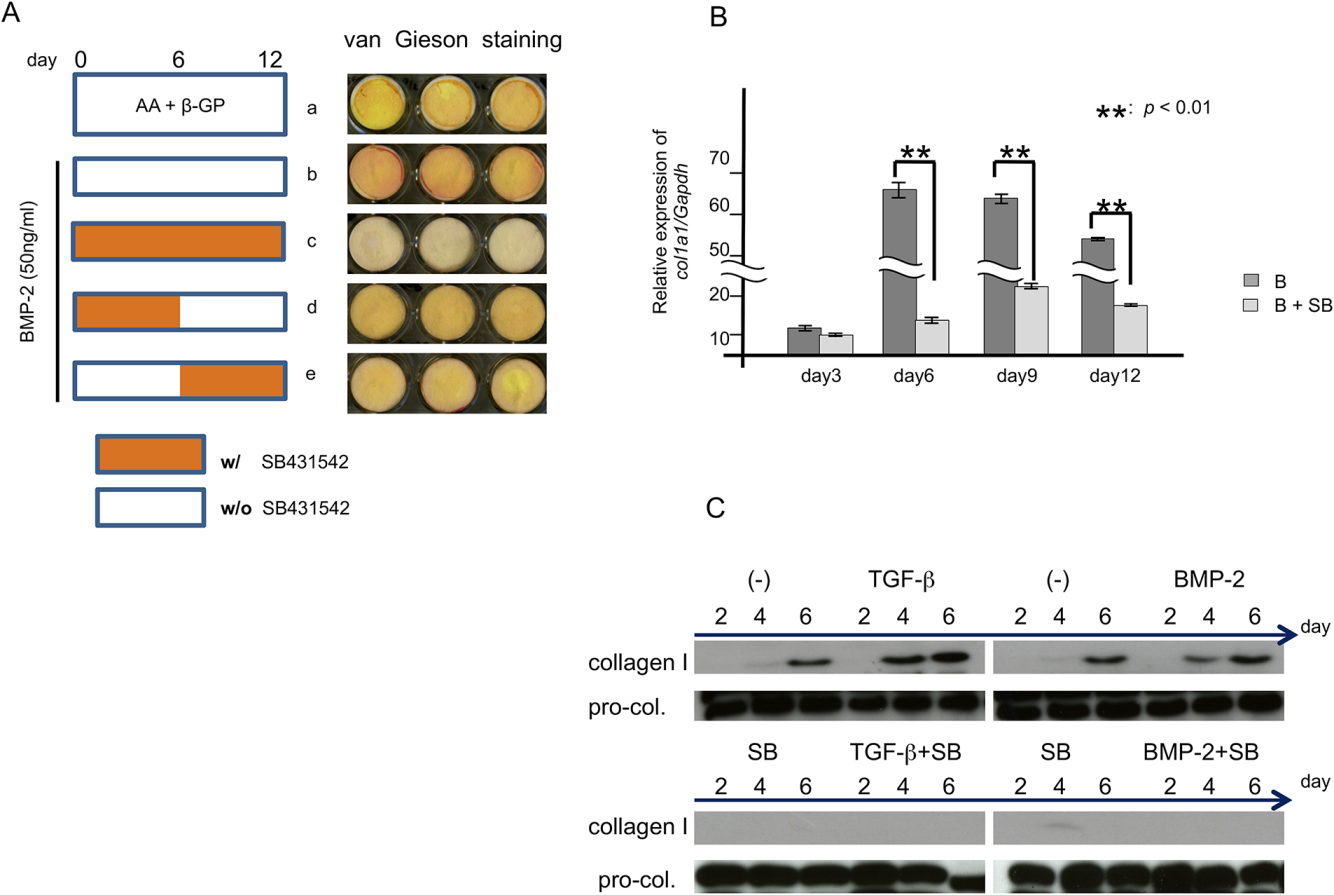
Effects of BMP-2 and SB431542 on collagen synthesis during osteoblastic differentiation of MPDL22 cells. (A) SB431542 (10 µM) was added to MPDL22 cells during osteogenic differentiation with or without BMP-2 (50 ng/mL) at different times as indicated in the left panel. The right panel shows the van Gieson staining, which stains collagen pink. (B) The relative quantification of *Col1A1* mRNA in BMP-2-induced MPDL22 cells was assessed during osteogenic differentiation in the presence or absence of SB431542 (10 μM). MPDL22 cells were harvested every 3 days and isolated mRNA was assessed by RT-qPCR. Quantitative mRNA values were normalized to the amount of *GAPDH* mRNA. B: BMP-2; SB: SB431542, **: p<0.01 vs BMP-2. (C) The protein synthesis of collagen I in MPDL22 cells was examined by western blotting. The culture supernatants were aspirated at the indicated time points from MPDL22 cells treated by BMP-2 (50 ng/mL) and TGF-β (4 ng/mL) in the presence or absence of SB431542 (10 μM) in long-term cultures. SB: SB431542.

Because collagen plays a crucial role as a constituent ECM molecule in hard tissue, we examined the effects of SB431542 on collagen synthesis in MPDL22 cells using van Gieson staining. Interestingly, SB431542 inhibited the BMP-2-stimulated formation of van Gieson-positive collagen fibers in the long-term cultures (Fig 6A). This inhibition of collagen synthesis was confirmed at both the transcript level by RT-qPCR analysis (Fig 6B) and the protein level by western blotting (Fig 6C). These results suggested that endogenous TGF-β is required for the synthesis and production of sufficient ECM proteins from MPDL22 cells.

## Discussion

### SB431542 as a TGF-β receptor inhibitor

We demonstrated the physiological functions of TGF-β in PDL cells by examining the molecular mechanisms that regulate cell proliferation and cytodifferentiation during the ossification process. To identify the functions of TGF-β, we introduced SB431542, a small molecule inhibitor of TGF-β type I receptors to block endogenous TGF-β signaling, because a commercially available TGF-β neutralizing antibody did not effectively inhibit endogenous TGF-β signaling activated by the three TGF-β isoforms TGF-β1, TGF-β2, and TGF-β3 in PDL cells [20]. SB431542 was discovered by screening a small chemical compounds library and was reported to inhibit TGF-β signaling in various cell types [21,22,23]. SB431542 is a cell-permeable chemical that antagonizes TGF-β receptor signaling by inhibiting ATP binding to the intracellular kinase domain of receptor I [14]. Several such molecular targeting drugs for the TGF-β signaling pathway have been developed and are currently in clinical trials for cancer therapy [24, 25].

### ALKs receptor expression on PDL cells

The expression level of ALK-3 and ALK-5 receptors in MPDL22 cells was higher than for other ALKs. These results suggested that MPDL22 cells mainly use ALK-5 as a type I receptor for TGF-β and ALK-3 as type I receptor for BMP, along with other type II receptors in the TGF-β/BMP signaling cascades in PDL cells. A weak but significant expression of ALK-1 was detected in MPDL22 cells, which may activate disease-related Smad1-dependent signaling by TGF-β (Fig 1A). ALK-1 is expressed in endothelial cells, chondrocytes, and synoviocytes [26, 27, 28] and might be involved in the pathogenesis of hypertension and rheumatoid arthritis. We infer that ALK-1 may be involved in TGF-β-dependent cellular responses in PDL cells during SB431542 treatment and the analysis of ALK-1 function might provide further insights into the role of TGF-β signaling in PDL cells.

### Effects of a TGF-β receptor inhibitor on PDL cells

We also examined the role of endogenous TGF-β on the proliferation of MPDL22 cells. Exogenous FGF-2 stimulated MPDL22 cell proliferation in a dose-dependent manner. In contrast, exogenous TGF-β, even at 10 ng/mL, did not alter cell proliferation (S1 Fig).

During the cell proliferation phase, the addition of SB431542 did not strongly affect the response of MPDL22 cells. Indeed, SB431542 treatment inhibited cell growth under BMP-2 treatment (S1 Fig). This result may be caused by the inhibition of TGF-β production by BMP-2 (Fig 1D). Nevertheless, these results indicate that the function of TGF-β in mineralized tissue formation may not be through the clonal expansion of PDL stem cells, but involves MPDL22 cells.

Conversely, the role of TGF-β signaling was pivotal during the formation of mineralized tissues by MPDL22 cells (Fig 3). The inhibition of endogenous TGF-β signaling by SB431542 treatment enhanced the ALP activity and mineralized nodule formation in BMP-2-stimulated MPDL22 cells, as well as accelerated the expression of osteoblastic differentiation related genes, such as *ALPase*, *Runx2* and *BSP* (Figs 3 and 4). Notably, SB431542 treatment was not effective in enhancing MPDL22 ossification without BMP-2 stimulation. This result supports the idea that SB431542 targets the BMP-2-induced molecular or cellular events in MPDL22 cells. Consistent with these findings, SB431542 treatment was more effective during the BMP-2-induced early ossification period (days 0–4) than any other culture period (Fig 4). This suggests a potential regulation mechanism by TGF-β in the BMP-2 signal-dependent early commitment of MPDL22 cells into osteoblastic cells during this period. Moreover, SB431542 treatment partially suppressed the expressions of Smurf1, Smad6 and Smad7 during the late ossification period (days 9–12) (S2 Fig). These results suggest the dual functions of SB431542 in BMP-2-induced calcified nodule formation at different maturation stages. Smad7 inhibits both BMP and TGF-β/activin signaling, whereas Smad6 preferentially inhibits BMP signaling [7, 8]. This process is tightly regulated by other signaling components, including Smad ubiquitination regulatory factors (Smurfs), particularly Smurf1 and Smurf2. The Smurfs themselves, or in complexes with I-Smads, target R-Smads and TGF-β/BMP receptors for protein degradation by the ubiquitin-proteasome system [9, 10]. Smurf1 is an E3 ubiquitin ligase that binds PPXY motifs in target proteins, such as BMP-activated Smads 1 and 5 and Runx2. Genetic knockout animal models have revealed the physiological functions of Smurfs in skeletal development [29]. Recently, high levels of Smurf1 expression were reported in human PDL stem-like cells in chronic periodontal disease patients [30]. A previous study also reported Smad6 was a candidate molecular target for SB431542 in a mesenchymal stem cell differentiation model [31]. In the present study, we showed that the inhibition of endogenous TGF-β signaling resulted in blocking the negative feedback signaling cascades for the TGF-β/BMP-2 signaling pathway. Therefore, SB431542 treatment may participate in both osteoblastic differentiation and maturation of mineralized tissue formation in MPDL22 cells.

TGF-β induced collagen synthesis in MPDL22 cells during the ossification process and SB431542 treatment diminished the production of collagen I at the transcription and protein levels (Fig 6). Consistent with this result, the addition of SB431542 during the late ossification period decreased BMP-2-stimulated calcified nodule formation. However, the inhibition of collagen synthesis by SB431542 did not affect the enhancement of BMP-2-induced MPDL22 ossification (Fig 5). This suggests that BMP-2-induced collagen production is sufficient for the maintenance of mineralized tissue formation in MPDL22 cells. These results also suggest that endogenous TGF-β signaling may inhibit the BMP-2-induced early commitment of cells to osteoblastic cells, but positively regulate the ECM production that maintains the elastic properties of PDL tissues and sustains collagen synthesis for the maturation of ECM proteins towards mineralized tissue formation.

Recently, several small molecule inhibitors of TGF-β type I receptors have been developed. LY364947 and Inhibitor II are two such small molecules that inhibit TGF-β type I receptor, as well as ALK-4, -5, and -7. Both of these drugs enhanced the ossification of BMP-2-stimulated MPDL22 cells, though at different magnitudes (Fig 5). This result strongly supports the idea that the pharmacological inhibition of TGF-β signaling might enhance the BMP-2-induced formation of mineralized tissues in MPDL22 cells.

### Effects of SB431542 on hPDL cells

The exact function of TGF-β in hard-tissue formation remains ambiguous [32]. Systemic administration of a TGF-β receptor kinase inhibitor into an animal model showed anabolic and anti-catabolic effects on bone [33]. These results may be caused by the normal functions of the TGF-β family signaling molecules in cells and organs in a spatiotemporal manner. Nevertheless, the specific inhibition of a TGF-β type I receptor with a pharmacologic inhibitor showed an induction of cytodifferentiation of mesenchymal stem cells, chondrocytes, and angiogenesis [31,34,35]. Therefore, the regulation of TGF-β signaling is a promising approach for enhancing the regeneration of cells and organs in mineralizing tissues.

To explore the possibility of applying this chemical for clinical use, we also examined the effects of SB431542 on human (h)PDL cells [36]. We used three different primary hPDL cell lines that were established from the embedded incisor teeth of different patients. The application of SB431542 to the hPDL cultures clearly enhanced the calcified nodule formation induced by BMP-2. This result suggests that TGF-β signaling is also prominent in the ossification process of hPDL cells *in vitro* (S3 Fig). Notably, the effects of SB431542 on mineralized tissue formation were different in each hPDL cell line. This may reflect the different response characteristics of each hPDL cell line to TGF-β or the existence of varied drug sensitivity mechanisms among different individuals [37].

In conclusion, this study demonstrated that endogenous TGF-β prevented PDL cells from differentiating into hard tissue forming cells, such as osteoblasts and cementoblasts, during the early commitment stage by competing with the effects of BMP-2. TGF-β was also necessary for collagen synthesis by PDL cells during the late ossification stage. These results suggest that the application of a synthetic TGF-β inhibitor during the early phase may support the wound healing and regeneration of periodontal defects. This approach indicates that using small chemical compounds in combination with current cytokine therapies may stimulate the process of regeneration of periodontal tissue that is being destroyed by periodontal disease.

## Materials and Methods

### Ethics statement

All procedures involving human specimens were performed under written informed consent according to the Declaration of Helsinki and the protocol was approved by the ethical committee of Osaka University Dental Hospital.

### Reagents

TGF-β, BMP-2, PDGF-BB (R&D Systems, Minneapolis, MN, USA), FGF-2 (Stemgent, Cambridge, MA, USA), and TGF-β receptor inhibitors SB431542 (Tocris Bioscience, Bristol, UK), LY364947 (Sigma-Aldrich, St. Louis, MO, USA), and Inhibitor II (Merck, Darmstadt, Germany) were applied to cell culture media at the concentrations indicated.

### Cell culture of PDL cell line

Mouse PDL cell line MPDL22 was isolated from the PDL tissue of the molar teeth of BALB/c mice at 2.5 weeks of age and maintained as previously described [17]. Briefly, the donor MPDL22 cell was selected because it had the highest ALPase activity of the clonal mouse PDL cell lines that were obtained via limiting dilution. hPDL cells were isolated as described previously [38]. hPDL cells were obtained from the PDL tissue of impacted incisor teeth from three different donors. hPDL cells at passage numbers less than six were used in these experiments. MPDL22 and hPDL cells were cultured in α-modified Eagle's medium (α-MEM) supplemented with 10% fetal calf serum (FCS), 50 units/mL penicillin G, and 50 μg/mL streptomycin at 37°C under 5% CO_2_.

### ALP activity

To induce ossification, MPDL22 cells were cultured in α-MEM with 10% FCS in the presence of 10 mM GP and 50 μg/mL AAs (mineralization inducing medium) that was replaced every 3 days. ALPase activity was assessed according to the procedure of Bessey et al. [39]. Briefly, after washing twice with PBS, the cells were homogenized in a glass homogenizer in 1 mL of 0.9% NaCl with 0.2% Triton X-100 at 4°C and then centrifuged for 15 min at 12,000 ×g. ALPase activity in the supernatant was measured using p-nitrophenyl phosphate as the substrate. The supernatant was mixed with 0.5 M Tris-HCl buffer (pH 9.0) containing 0.5 mM p-nitrophenyl phosphate and 0.5 mM MgCl_2_. Next, the samples were incubated at 37°C for 30 min, and the reaction was stopped by the addition of 0.25 mL of 1 N NaOH. Hydrolysis of p-nitrophenyl phosphate was monitored on a spectrometer as a change in A410, and p-nitrophenol was used as a standard. One unit of activity was defined as the enzyme activity that hydrolyzed 1 nmol of p-nitrophenyl phosphate in 30 min.

### Mineralization of PDL cells

The histochemical staining of calcified nodules was performed following the modified Alizarin red staining method described by Dahl [40]. To induce ossification, MPDL22 and hPDL cells were cultured in mineralization-inducing medium that was replaced every 3 days. Briefly, the cell monolayers were washed twice with PBS and then fixed in dehydrated ethanol for 10 min. After fixation, the cells were stained with 1% Alizarin red S (Wako) in 0.1% NH_4_OH (pH 6.5) for 5 min. The wells were then washed with water and digitally scanned (GT-9700F, Epson, Suwa, Japan). The density of calcified nodules in each well was calculated using Win ROOF software (Mitani, Fukui, Japan). Positive scores were estimated by multiplying each stained area by its color density.

### Cell proliferation assay

The ratio of cell proliferation of MPDL22 cells was determined using bromodeoxyuridine (*BrdU*) incorporation assay kits (Roche Diagnostics, Indianapolis, IN, USA) according to the manufacturer’s instructions. The color change of the substrate was monitored on a microplate luminometer (GloMax-96, Promega, Madison, WI, USA) at an optical density of 420 nm.

### ELISA assay for TGF-β1 production

TGF-β release into the culture supernatants by MPDL22 cells was measured using an ELISA kit for mouse TGF-β (R&D Systems) according to the manufacturer’s instructions. Culture supernatants from MPDL22 cells stimulated by BMP-2 and cultured with or without SB431542 were used for the assay.

### Luciferase assay

MPDL22 cells were transiently co-transfected with 12× (*CAGA)*-Luc luciferase vector [10] and pRL-TK renilla luciferase vector (Promega) by Lipofectaine2000 (Life Technologies, Carlsbad, CA, USA). After 24 h, transfected cells were pre-treated with or without SB431542, and then stimulated with 4 ng/mL of TGF-β1 for the following 18 h. Luciferase activity was determined using the Dual luciferase reporter assay system on the microplate luminometer (Promega).

### Western blotting and analysis

Prior to treatment with TGF-β1 (4 ng/mL, R&D), BMP-2 (50 ng/mL, R&D), FGF-2 (20 ng/mL, Stemgent) or SB431542 (10 µM, Tocris) for the indicated time, cells were starved overnight in α-MEM containing 0.2% FCS. After stimulation and two washes with ice cold PBS, cells were harvested in RIPA lysis buffer (Millipore, Billerica, MA, USA) with protease and phosphatase inhibitors. The protein concentrations of the cell lysates were determined by the Bradford method (Bio-Rad, Hercules, CA, USA). Primary antibodies included anti-phospho-Erk, anti-phospho-p38, anti-Erk1, anti-p38, and anti-phospho-Smad3 (1:2000, Cell Signaling, Danvers, MA, USA); anti-Smad2/3 (1:2000, BD Biosciences, San Jose, CA, USA); anti-BMP type I receptor, anti-BMP type II receptor, anti-TGF-β type I receptor, and anti-TGF-β type II receptor (1:500, Santa Cruz, Santa Cruz, CA, USA); anti-collagen I (Millipore); and anti-β-actin (1:5000, Sigma-Aldrich). Secondary antibodies included horseradish peroxidase (HRP)-conjugated sheep anti-mouse IgG and HRP-conjugated donkey anti-rabbit IgG (1:5000, Cell Signaling).

### RT-PCR and RT-qPCR analyses

RNA was prepared with Trizol (Life Technologies) according to the manufacturer’s instructions. Total RNA extract was reverse-transcribed with the High Capacity RNA-to-cDNA Kit (Applied Biosystems Instruments (ABI), Foster City, CA, USA). Conventional RT-PCR was performed by DNA engine (Bio-Rad) with gene-specific primers (S1 Table). PCR products were analyzed by agarose gel electrophoresis. Semiquantitative q-PCR was performed using an ABI 7300 Fast Real-Time PCR System with Power SYBR Green PCR Master Mix (ABI) and gene-specific primers (S1 Table) according to the manufacturer’s instructions. The relative expression is shown after normalization to glyceraldehyde-3-phosphate dehydrogenase (*GAPDH*) expression.

### Statistical Analysis

All of the experiments in this study were performed at least three times. The data presented are representative of all results. Data are presented as the mean and standard deviation of triplicate assays. The statistical significance of the differences between two means was assessed using Student’s t-tests for paired comparisons or one-way analysis of variance for multiple comparisons with Bonferroni post-hoc tests. Values of p less than 0.05 were considered to indicate significant differences.

## Supporting Information

S1 Fig Effects of endogenous TGF-β on MPDL22 cell proliferation. The effects of TGF-β, FGF-2 and BMP-2 in the presence or absence of SB431542 on the proliferation of MPDL22 cells **(A)** MPDL22 cells were cultured with FGF-2 (10 or 100 ng/mL) or TGF-β (4 ng/mL) for 24 h. **: p<0.01 vs FGF-2 (10 ng/mL) **(B)** The effect of SB431542 (100 μM) on the BMP-2-induced proliferation of MPDL22 cells. *BrdU* incorporation rates were measured using a luminometer at an absorbance of 420 nm. **: p<0.01 vs BMP-2.
(TIF)

S2 Fig Smad and Smurfs mRNA expression in MPDL22 cells during osteogenic differentiation. The relative quantification of *Smad6*, *Smad7*, *Smurf1* and *Smurf2* mRNAs during osteogenic differentiation of MPDL22 cells by BMP-2 (50 ng/mL) after treatment in the presence or absence of SB431542 (10 μM) for 3 days. MPDL22 cells were harvested every 3 days, and the isolated mRNA was assessed by RT-qPCR. Quantitative mRNA values were normalized to the amount of *GAPDH* mRNA. B: BMP-2; SB: SB431542, **: p<0.01 vs BMP-2; *: p<0.05 vs BMP-2.
(TIF)

S3 Fig Effects of SB431542 on the osteoblastic differentiation of hPDL cells. SB431542 (10 μM) was added to three hPDL cell lines during BMP-2-induced osteogenic differentiation. Calcified nodule formation was determined by Alizarin red staining at days 24, 27 and 30. B: BMP-2; SB: SB431542,
(TIF)

S1 Table Primers used in the present study. (PDF)
