# Supplementary figures and images for: TGF-Beta Negatively Regulates the BMP2-Dependent Early Commitment of Periodontal Ligament Cells into Hard Tissue Forming Cells
Source: PLoS One. 2015 May 13;10(5):e0125590. doi: 10.1371/journal.pone.0125590 (PMC4430433; doi:10.1371/journal.pone.0125590)

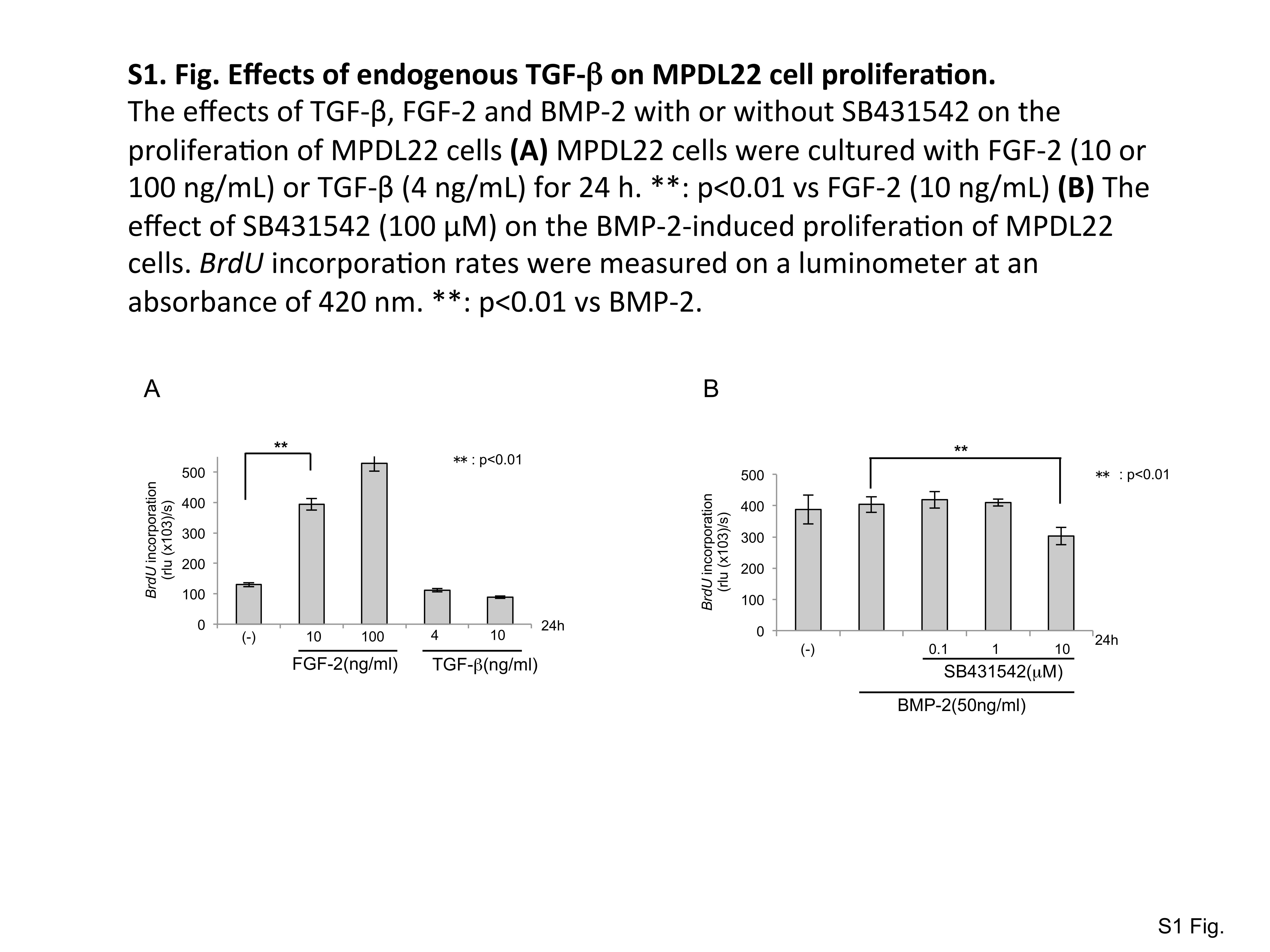

Supplement: S1 Fig — The effects of TGF-β, FGF-2 and BMP-2 in the presence or absence of SB431542 on the proliferation of MPDL22 cells (A) MPDL22 cells were cultured with FGF-2 (10 or 100 ng/mL) or TGF-β (4 ng/mL) for 24 h. **: p<0.01 vs FGF-2 (10 ng/mL) (B) The effect of SB431542 (100 μM) on the BMP-2-induced proliferation of MPDL22 cells. BrdU incorporation rates were measured using a luminometer at an absorbance of 420 nm. **: p<0.01 vs BMP-2. (TIF) [file pone.0125590.s001.tif]

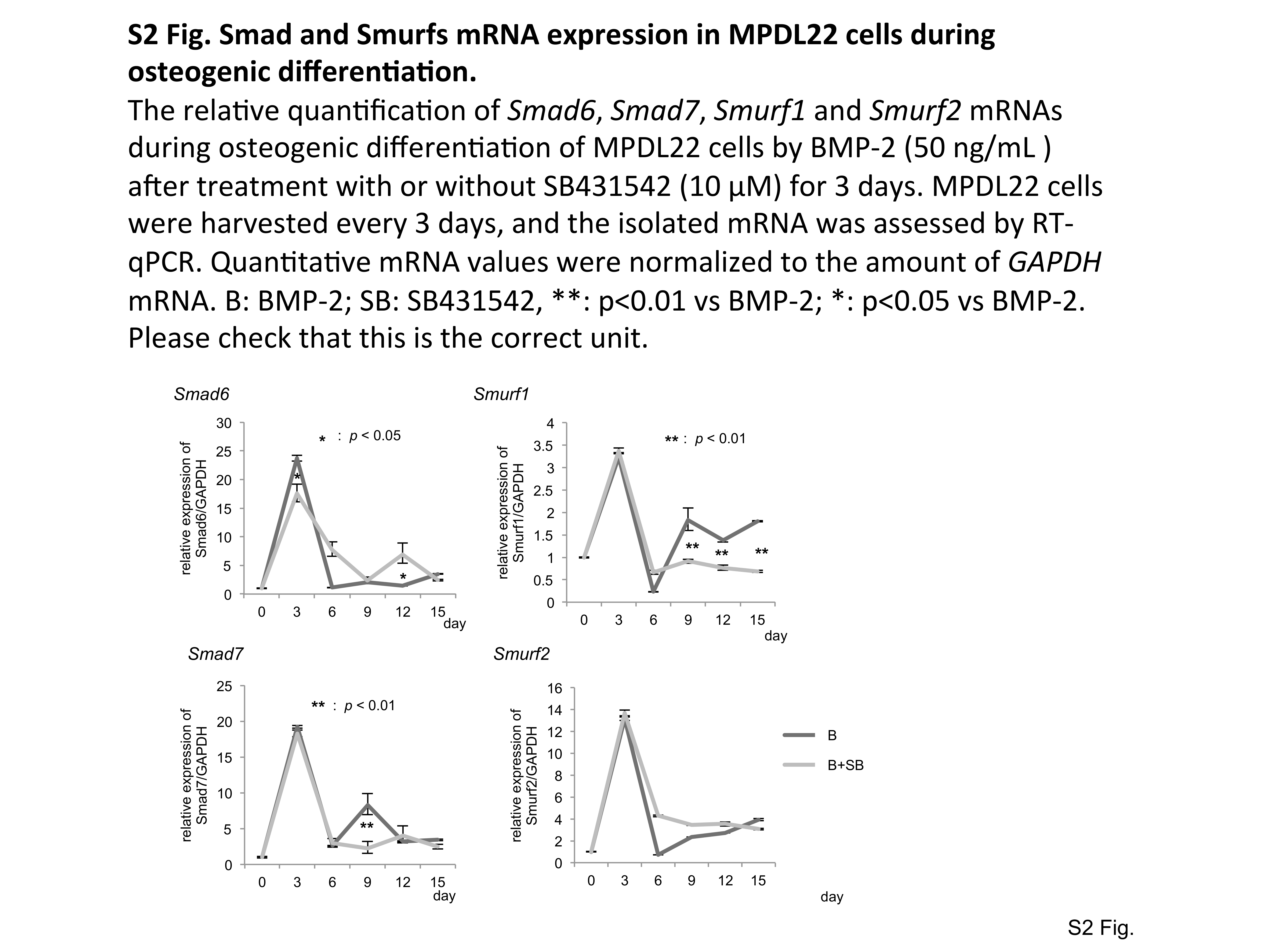

Supplement: S2 Fig — The relative quantification of Smad6, Smad7, Smurf1 and Smurf2 mRNAs during osteogenic differentiation of MPDL22 cells by BMP-2 (50 ng/mL) after treatment in the presence or absence of SB431542 (10 μM) for 3 days. MPDL22 cells were harvested every 3 days, and the isolated mRNA was assessed by RT-qPCR. Quantitative mRNA values were normalized to the amount of GAPDH mRNA. B: BMP-2; SB: SB431542, **: p<0.01 vs BMP-2; *: p<0.05 vs BMP-2. (TIF) [file pone.0125590.s002.tif]

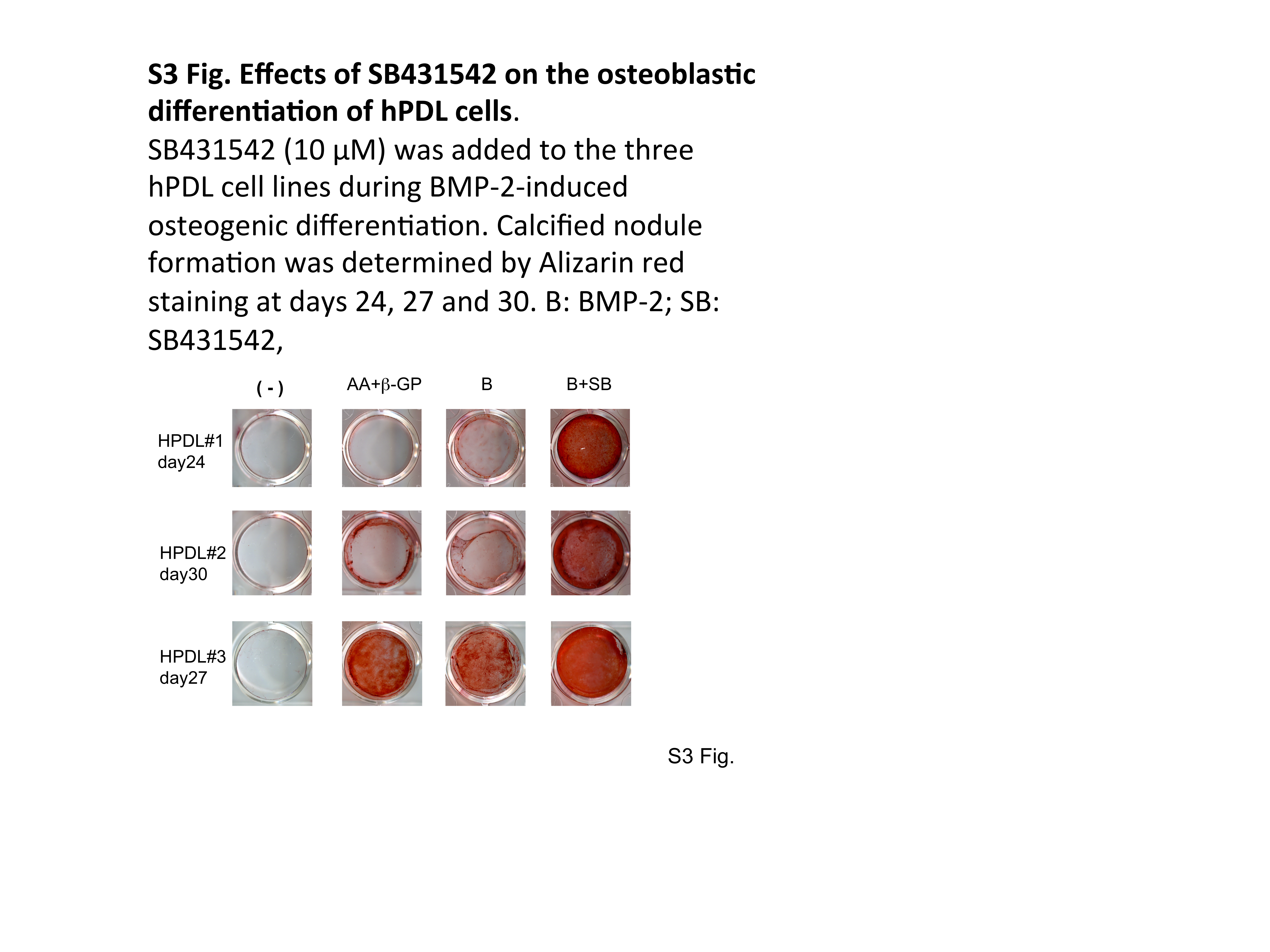

Supplement: S3 Fig — SB431542 (10 μM) was added to three hPDL cell lines during BMP-2-induced osteogenic differentiation. Calcified nodule formation was determined by Alizarin red staining at days 24, 27 and 30. B: BMP-2; SB: SB431542, (TIF) [file pone.0125590.s003.tif]
