# Supplementary material for: TGF-Beta Negatively Regulates the BMP2-Dependent Early Commitment of Periodontal Ligament Cells into Hard Tissue Forming Cells
Source: PLoS One. 2015 May 13;10(5):e0125590. doi: 10.1371/journal.pone.0125590 (PMC4430433; doi:10.1371/journal.pone.0125590)
Supplement: S1 Table — (PDF) [file pone.0125590.s004.pdf]

S1 Table. Primers used in this study

A. For RT-PCR

gene		
Alk1	Primer—Forward	5'- TGACTTTCTGCAGAGGCAGA -3'
	Primer—Reverse	5'- CGACTCAAAGCAGTCTGTGC -3'
Alk2	Primer—Forward	5'- ATGACTACCTTCAGCTCACT -3'
	Primer—Reverse	5'- CTTCGCCAGAGAAGTTAATG -3'
Alk3	Primer—Forward	5'- TACACTGCCCCCTGTTGTTA -3'
	Primer—Reverse	5'- CTCTGGTGTCTAGTGTGGCA -3'
Alk4	Primer—Forward	5'- ACCGCTACACAGTGACCATT -3'
	Primer—Reverse	5'- TCTTCACATCTTCCTGCACG -3'
Alk5	Primer—Forward	5'- ATCCATCACTAGATCGCCCT -3'
	Primer—Reverse	5'- CGATGGATCAGAAGGTACAAGA -3'
Alk6	Primer—Forward	5'- CACCTTAGACGCAAAGTCCA -3'
	Primer—Reverse	5'- CCATCGATTGGGGAATGAA -3'
Alk7	Primer—Forward	5'- AGCTCGAAGGTGTTCAGTTGGA -3'
	Primer—Reverse	5'- AGTCAAGACCTGCCCATGCTTA -3'
Gapdh	Primer—Forward	5'- ATGTGTCCGTCGTGGATCTGA -3'
	Primer—Reverse	5'- TTGAAGTCGCAGGAGACAACCT -3'
Smad1	Primer—Forward	5'- ACTGAAGCCTCTGGAATGCT -3'
	Primer—Reverse	5'- GCGGTTCTTATTGTTGGACG -3'
Smad2	Primer—Forward	5'- CGGAGATTCTAACAGAACTG -3'
	Primer—Reverse	5'- TGCTTGAGCATCGCACTGAA -3'
Smad3	Primer—Forward	5'- AGCACACAATAACTTGGACC -3'
	Primer—Reverse	5'- TAAGACACACTGGAACAGCGGATG -3'
Smad4	Primer—Forward	5'- CATTCCTGTGGCTTCCACAA -3'
	Primer—Reverse	5'- GACTGATGGCTGGAGCTATT -3'
Smad5	Primer—Forward	5'- ATGCCCAGCATATCCAGCAG -3'
	Primer—Reverse	5'- CAGAAGAAATGGGGTTCAGC -3'
Smad6	Primer—Forward	5'- TTGCAACCCCTACCACTTCAGC -3'
	Primer—Reverse	5'- AGAATCGGACAGATCCAGTGGC -3'
Smad7	Primer—Forward	5'- TTGCCTCGGACAGCTCAATT -3'

	Primer—Reverse	5'- TGCTGCGGTTGTAAACCCA -3'
Bmpr2	Primer—Forward	5'- AGATCTATCCTCTCCCTAAG -3'
	Primer—Reverse	5'- TTAGAATGGACTGCCCTGTC -3'
Tgfbr2	Primer—Forward	5'- CCCCGGAAACTGATGGATTT -3'
	Primer—Reverse	5'- AGCTCCGTGTTGTGGTTGATGT -3'

B. RT-qPCR

Smad6	Primer—Forward	5'- TTGCAACCCCTACCACTTCAGC -3'
	Primer—Reverse	5'- AGAATCGGACAGATCCAGTGGC -3'
Smad7	Primer—Forward	5'- TTGCCTCGGACAGCTCAATT -3'
	Primer—Reverse	5'- TGCTGCGGTTGTAAACCCA -3'
Smurf1	Primer—Forward	5'- CTACCAGCGTTTGGATCTAT -3'
	Primer—Reverse	5'- TGTCTCGGGTCTGTAAACT -3'
Smurf2	Primer—Forward	5'- CAGCACCTGCTGAAGACATTTGA -3'
	Primer—Reverse	5'- TCCAGAACCACTTGACGACATTG -3'
Al	Primer—Forward	5'- ACACCTTGACTGTGGTTACTGCTGA -3'
	Primer—Reverse	5'- CCTTGTAGCCAGGCCCGTTA -3'
Runx2	Primer—Forward	5'- CACTGGCGGTGCAACAAGA -3'
	Primer—Reverse	5'- TTTCATAACAGCGGAGGCATTTC -3'
osterix	Primer—Forward	5'- CGCATCTGAAAGCCCACTTG -3'
	Primer—Reverse	5'- CAGCTCGTCAGAGCGAGTGAA -3'
Bsp	Primer—Forward	5'- ATGGAGACTGCGATAGTTCCGAAG -3'
	Primer—Reverse	5'- CGTAGCTAGCTGTTACACCCGAGAG-3'
Msx2	Primer—Forward	5'- GAAGACGGAGCACCGTGGATA -3'
	Primer—Reverse	5'- TCCAAGGCTAGAAGCTGGGATG -3'
Gapdh	Primer—Forward	5'- TGTGTCCGTCGTGGATCTGA -3'
	Primer—Reverse	5'- TTGCTGTTGAAGTCGCAGGAG -3'
